# Tracking protein turnover and degradation by microscopy: photo-switchable versus time-encoded fluorescent proteins

**DOI:** 10.1098/rsob.140002

**Published:** 2014-04-16

**Authors:** Michael Knop, Bruce A. Edgar

**Affiliations:** Zentrum für Molekulare Biologie der Universität Heidelberg, Deutsches Krebsforschungszentrum, DKFZ–ZMBH Allianz, Im Neuenheimer Feld 282, 69120 Heidelberg, Germany

**Keywords:** protein dynamics, fluorescent timer proteins, live cell microscopy, photo-switchable fluorescent proteins

## Abstract

Expanded fluorescent protein techniques employing photo-switchable and fluorescent timer proteins have become important tools in biological research. These tools allow researchers to address a major challenge in cell and developmental biology, namely obtaining kinetic information about the processes that determine the distribution and abundance of proteins in cells and tissues. This knowledge is often essential for the comprehensive understanding of a biological process, and/or required to determine the precise point of interference following an experimental perturbation.

## 2. Introduction

In an ideal world the researcher could simply follow each individual protein molecule under study with exact spatial and temporal resolution—tracking single polypeptides from birth (by translation), throughout their lives to death (by degradation). But this is currently impossible. At present, the only way to obtain the parameters that impact protein lifetimes is to track hundreds, thousands or even millions of proteins in bulk, recording mean parameter estimates.

**Figure 1.**
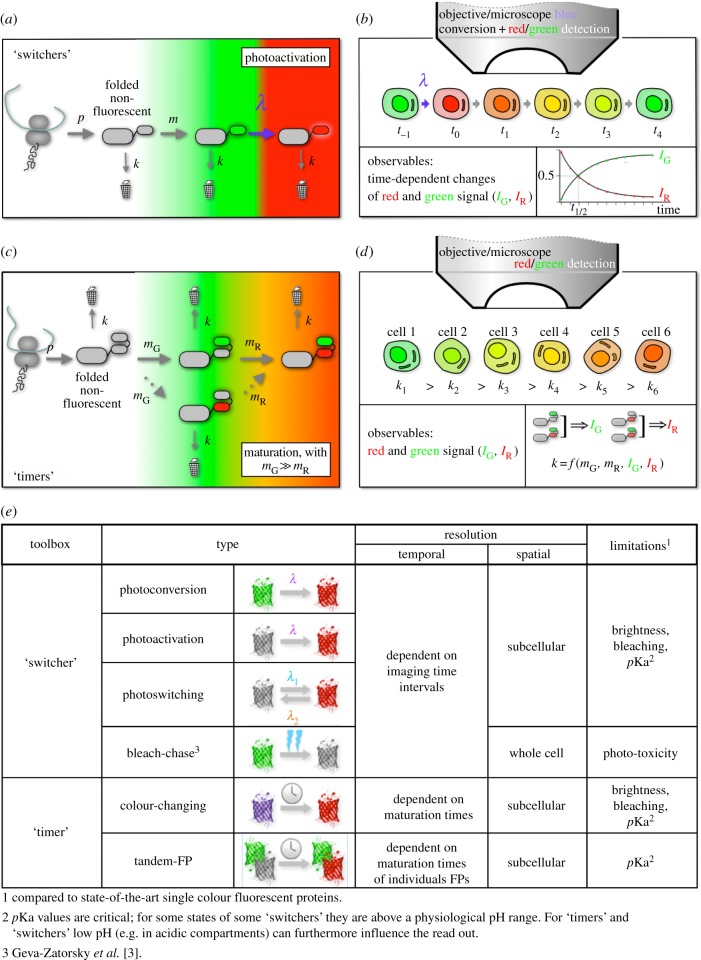
Application of FPs to quantify protein turnover and degradation. (*a*) FPs that change their spectral properties as a function of a light intervention (here generally termed ‘switcher’; the example of a green-to-red photoconvertible FP is given) can be used in pulse-chase types of experiments. Depending on the properties of the FP, which can be either photoactivatable, photoswitchable or photoconvertable [4], a pool of labelled proteins is generated using illumination with light of a specific wavelength and intensity. In the simplest scenario as depicted here (*b*), the speed with which un-marked proteins replace marked ones (as observed during the chase period) is quantified. Assuming a steady-state situation, in which protein production and degradation (*k*) are constant, the half-life of the protein (*t*_1/2_) can be directly estimated [2]. The illustration depicts whole cell measurements; however, sub-cellular measurements to determine local turnover are possible, limited only by the number of available fluorophores and their particular brightness and photobleaching properties. (*c*) Fluorescent timer proteins can be categorized into two groups: single FPs that change their colour as a function of time (owing to subsequent chemical reactions that lead to changes in the fluorophore), and tFTs. Both types report on the average age of a pool of proteins. (*d*) In its simplest application under steady-state conditions, the average age of the proteins directly reports on protein degradation rates (‘fast degrading proteins die young’), independent of the protein production rates [5]. Tandem FP timers use a fast maturing FP such as superfolder GFP as a reporter for protein abundance, while a slow maturing protein, i.e. an RFP, reports on the relative age of the GFP marked pool. Tuning of the dynamic range here is achieved by choosing RFPs with an appropriate maturation time. (*e*) Table listing properties of different ‘switcher’ FPs and some considerations for their application to conduct degradation/turnover measurements.

To do this, two experimental fluorescent protein (FP)-based toolkits are available. The first of these employs microscopy-based pulse-chase type experiments [1–3] (figure 1*a,b*,*e*). Here, photo-switchable FPs that change, acquire or lose fluorescence as a function of an intervention [4,6] are the most practical. A pulse of local light-irradiation generates a labelled or activated population of a protein species; this population is then followed in time and space. With this ‘switcher’ method, the temporal component of a measured turnover constant must necessarily be obtained from the time course parameters, upon fitting of measured signal intensities to models that describe the process, thus yielding turnover estimates.

The second toolkit employs fluorescent timers: these FPs change their colour as a function of protein age (figure 1*c*–*e*), owing to fluorophore maturation kinetics, determined by stochastically occurring chemical reactions. Here, time information is intrinsically encoded in the FP reporter. The relative read-out of signal intensities from the two different colour states of the FP at a single-time point instantaneously reports on the average age of the observed protein pool [7]. Because single-time point imaging is informative in this case, live-imaging may even be dispensable for some applications, allowing the use of fixed, prepared samples and higher resolution imaging, e.g*.* from cells, organs or tissue sections isolated from a living animal. With FP timers, turnover rate constants can be inferred by fitting the data to models that already contain time information, based on *a priori* knowledge of the maturation kinetics of the FP probes. Importantly, under steady-state conditions of constant protein synthesis and degradation, these models do not need to consider the synthesis rate, therefore allowing the researcher to retrieve comparable estimates for degradation rates of different proteins.

Because of the rapid development of instrumentation for super-resolution microscopy, photo-switchable FPs have received particular attention. Consequently, a broad range of new proteins with optimized properties is available, close to the performance limits that FPs are theoretically able to deliver. Fluorescent timers have received comparatively less attention since the initial, highly anticipated description of colour-changing FPs. But progress has been made in developing better performing proteins [8], and the recent development of a new class of fluorescent timers, consisting of tandem FP fusions (tFTs), seems to have overcome the major limitations of the original timers in which colour changes were encoded in just one FP domain. This new generation of timers uses tandem fusions of conventional FPs, each moiety with a different maturation time [5]. Such tFTs benefit directly from the development of super-bright, optimally ‘behaving’ ‘normal’ FPs.

A recent study using zebrafish provides a particularly striking illustration of the potential of tFTs for visualizing protein turnover in a living organism, requiring nothing more than a standard epi-fluorescence microscope with two-colour imaging. Using a G-protein-coupled receptor (GPCR) tagged with a tFT, Dona *et al*. [9] demonstrated that the timer read-out can report the age and thereby the turnover of the GPCR at the plasma membrane. The GPCR-tFT proved to be a sensitive, reliable tool for mapping cytokine signalling activity in a rather large collective of migrating cells, which in this case established a ‘standing wave’ with higher receptor activity at one side of the cell collective, and lower signalling at the other. The standing wave of signalling activity gave the cell collective an intrinsic polarity that could not have been easily detected by other means. Importantly, imaging the tFT was possible in whole living embryos at cellular resolution, for long periods of time, without any physical intervention.

Both toolkits—switchers and timers—exhibit intrinsic advantages and limitations that dictate their ability to report on protein dynamics. These depend on culture conditions, the organismal or cellular context, and available instrumentation. While it is not our intent to discuss these details here, a few general considerations are of utmost importance and require careful consideration. First, both toolkits are limited by the intrinsic properties of FPs (partially listed in figure 1*e*). One intrinsic limitation is that FPs require time to mature; while this is exploited for timers and often ignored for switchers, these parameters are not absolute constants, but probably depend on a protein's physico-chemical and folding environment. This implies that published values of specific FP properties are at most hints, and need to be re-measured in the system of interest to calibrate either a switcher or a timer. If not matured, the fluorophore cannot be observed, and hence the observed population contains a ‘dark’ fraction of unknown quantity that may significantly influence the analysis.

Measurements using both switchers and timers are grossly simplified when the observed system is in steady state, as this requires simpler models with fewer parameters, and temporal changes within the system itself need not be considered. Out of steady-state processes (e.g. measurements during cellular transitions) are rather difficult to address using switchers, especially if the timescale of time course measurements is similar to or slower than the cellular transition under study. Here, timers are valuable, because they enable time-series recording of how a time-containing parameter (the age of a protein pool) evolves.

Another significant challenge is how exactly to extract the desired parameters (e.g. a protein's half-life or transport speed) from kinetic measurement data. Indeed, whether there might be a general approach applicable to all situations is still an open question. Often an essential ingredient to the solution might be found in computational simulations, typically using networks of coupled differential equations, or simply stochastic simulations. General models that initially contain all possible behaviours of a given protein can be built, and progressively narrowed and constrained as critical parameters are estimated from quantitative imaging with FP probes. In an iterative process, new experiments can be designed to retrieve additional parameters, for instance by experimentally altering or locking a known parameter, and thus the model can be constrained further. Necessary controls are largely dependent on the process under investigation, but certainly need to address the quality of the measurement (image quantification error) and the behaviour of the FPs (e.g*.* bleaching), and rule out toxic effects of the measurements on the cells.

Despite these complexities, fluorescent timers hold a lot of promise for the intracellular and intraorganismal imaging of protein dynamics. Many processes in cell biology, development and tissue homeostasis depend on temporal and spatial regulation, in which key molecules are subjected to differential expression and/or turnover control. This applies to basic cellular processes such as protein trafficking and secretion, as well as to virtually all signalling systems, from the simple two-cell interactions of yeast mating to collective tissue behaviours like the zebrafish example noted above. tFT timers might even be used at the physiological level, to measure for instance the turnover and delivery times of circulating blood serum factors. Because they do not require a physical intervention and can be imaged in single snap shots, tFP timers may have a wider range of potential application than photo switchers or photobleaching. In principle, they could yield valuable information not only about membrane-tethered receptors like GPCRs but also about nuclear receptors, transcription factors, guidance molecules, secreted signals, so-called ‘scaffolding proteins’, adhesion molecules and even extracellular matrix components [10]. From relatively crude genetics experiments and artificial cell culture assays, the stability of many such molecules is already believed to determine their activity and range of action, and hence the properties of the signalling systems in which they operate. But only in a few simplified scenarios has a quantitative analysis accurately measured the real *in vivo* dynamics of a signalling protein in action. tFP timers can in principle be used to measure the relative ages of a protein in different cellular compartments, or its occupancy time in macromolecular structures such as chromatin or stress granules. They should also be useful for determining the half-lives of different protein isoforms (e.g. phosphorylatable/non-phosphorylatable), identifying degradation motifs, defining parameters in source/sink gradients and (in time course experiments) determining time delays in relay networks following a stimulus. The future is indeed bright, all in red and green.
